# Associations between inflammation markers (CRP and IL-6) and depression among adolescents and young adults in Soweto and Durban, South Africa

**DOI:** 10.1016/j.bbih.2026.101265

**Published:** 2026-05-19

**Authors:** Tatiana E. Pakhomova, Thumbi Ndung'u, Mark Brockman, Anne Gadermann, T.J. Salway, Mags Beksinska, Amanda Rowlands, Kalysha Closson, Julie Jesson, Stepfanie Vermaak, Smritee Dabee, Janan J. Dietrich, Jenni Smit, Mzikazi Nduna, Angela Kaida

**Affiliations:** a Faculty of Health Sciences, Simon Fraser University, Burnaby, Canada; b HIV Pathogenesis Programme, The Doris Duke Medical Research Institute, University of KwaZulu-Natal, Durban, South Africa; c Africa Health Research Institute, Durban, South Africa; d Ragon Institute of Mass General Brigham, MIT and Harvard University, Cambridge, MA, United States of America; e Institute of Infection, Immunity and Transplantation, University College London, London, United Kingdom; f School of Population and Public Health, University of British Columbia, Vancouver, Canada; g Wits MRU (MatCH Research Unit), Department of Obstetrics and Gynaecology, Faculty of Health Sciences, University of the Witwatersrand, Durban, South Africa; h Univ Toulouse, Inserm, CERPOP, Toulouse, France; i Perinatal HIV Research Unit (PHRU), Faculty of Health Sciences, University of the Witwatersrand, Johannesburg, South Africa; j AVReQ, University of Stellenbosch, and the Human Sciences Research Council (HSRC), South Africa; k African Social Sciences Unit of Research and Evaluation (ASSURE), Faculty of Health Sciences, University of the Witwatersrand, Johannesburg, South Africa; l Health Systems Research Unit, South African Medical Research Council, Bellville, South Africa

**Keywords:** Adolescents, Young adults, Youth, CRP, IL-6, Inflammation, Depression, South Africa

## Abstract

**Objectives:**

Previous research has shown an association between inflammation and the development of depression. We examined the relationship between biomarkers of inflammation and probable depression among South African youth.

**Methods:**

We used cross-sectional data from 396 youth aged 16-24 from AYAZAZI, a cohort study (2014-2019) assessing socio-behavioural, structural, and biomedical HIV risk factors among youth. Depressive symptoms (past 7 days) were assessed using the 10-item Centre for Epidemiological Studies Depression (CES-D-10) Scale; ≥10 indicated probable depression. Inflammation was assessed using biomarkers C-Reactive Protein (CRP, in mg/L) and Interleukin-6 (IL-6, in pg/ml) which were quantified in plasma by Enzyme-Linked Immunosorbent Assays (ELISA). CRP and IL-6 concentrations were natural log transformed standardized prior to analysis and examined as continuous measures. Multivariable logistic regression models estimated associations between measures of inflammation and probable depression, adjusting for confounders. We tested effect modification by gender and age.

**Results:**

Among the 396 participants, 49.7% (n = 197) were aged 16-18, and 59.3% (n = 235) were women. Overall, 42.7% (n = 169) had probable depression. CRP was associated with 32% increased odds of probable depression in adjusted models (aOR 1.32; 95% CI 1.06-1.66); no significant association was found for IL-6 (aOR 1.06; 95% CI 0.85-1.32). Gender and age did not modify the relationship between CRP or IL-6 and probable depression.

**Discussion:**

Among South African adolescents and young adults, higher CRP, but not IL-6, was significantly associated with greater odds of probable depression. Findings align with growing evidence linking CRP to depression and contribute novel youth-focused data to the inflammation and mental health literature.

## Highlights

• 42.6% of South African youth reported probable depression.
• Higher plasma CRP concentration was significantly associated with greater odds of probable depression.
• Gender and age did not modify inflammation-depression associations.
• Study adds youth-focused data to inflammation and mental health research.

## 1 Introduction

Depression is a common and debilitating multicausal illness characterized by persistent feelings of sadness, loss of interest or pleasure, feelings of excessive guilt or low self-worth, disrupted sleep, changes in weight, and fatigue (World Health Organization, 2017). Depression often emerges in early adulthood, and has been shown to persist into adult life and contribute significantly to life-course morbidity, making it a leading global health issue (Kessler et al., 2005). These individual-level impacts underscore the importance of understanding depression's underlying mechanisms, particularly those that emerge early in life.

Growing evidence suggests that inflammation plays a role in the development of depression (Beurel et al., 2020). Experiences of chronic stress have been linked to dysfunction of the immune, nervous, and endocrine systems (Beurel et al., 2020; Mac Giollabhui, 2021; Liu and Alloy, 2010; Kiecolt-Glaser et al., 2015), impacting the body's ability to maintain homeostatic balance and regulate responses to various stress stimuli (Iob et al., 2020; Raison and Miller, 2017). Long-term psychological and physiological stressors can lead to the over-activation of immune responses, chronic inflammation and overall immune function dysregulation (Beurel et al., 2020). This persistent low-grade stimulation of inflammatory responses, and the over-production and continuous presence of acute inflammatory proteins such as cytokines (Furman et al., 2019; Maydych, 2019; Dowlati et al., 2010), are implicated in the etiology of numerous physiological and neurological diseases (Kautz, 2021; Liu et al., 2017; Boyce et al., 2021), including the onset and exacerbation of depression (Beurel et al., 2020; Mac Giollabhui, 2021; Liu and Alloy, 2010; Kiecolt-Glaser et al., 2015). Cytokines and other acute inflammatory proteins are modulators of nervous and immune system function and are important in the maintenance of neurological function, playing both pro- and anti-inflammatory roles (Furman et al., 2019; Maydych, 2019; Dowlati et al., 2010; Zhang and An, 2007; Tian et al., 2014), and can alter mood and cognitive function (Beurel et al., 2020; Dantzer et al., 2007, 2008). Consistently, elevated levels of inflammatory biomarkers have been found in individuals with major depressive disorder, and as such have been investigated in the context of depressive illness (Beurel et al., 2020; Haapakoski et al., 2015; Osimo et al., 2019).

Among these biomarkers, C-reactive protein (CRP) and interleukin-6 (IL-6) have received particular attention. Systematic reviews and meta-analyses indicate moderate to strong evidence for associations between major depression and IL-6, and CRP, with less significant but present evidence for tumor necrosis factor-α (TNF-α) levels and interleukin-1β (IL-1β) (Haapakoski et al., 2015; Osimo et al., 2019; Valkanova et al., 2013). While both CRP and IL-6 are involved in the acute phase responses to immune activation, they operate through distinct pathways (Del Giudice and Gangestad, 2018). IL-6, produced by macrophages, regulates immune function responses to immune challenges such as injury or infection, promoting B and T cell production (Tanaka and Kishimoto, 2014; Ferrucci et al., 2005). CRP, whose expression is induced by IL-6, is mainly produced by the liver in response to immune activation. Both molecules are commonly used as clinical markers for investigating chronic inflammation (Del Giudice and Gangestad, 2018; Calder et al., 2013; Nehring et al., 2017).

Importantly, depression and inflammation share several social and structural risk factors (Beurel et al., 2020; Slavich and Irwin, 2014a; Slavich and Sacher, 2019). Research strongly supports the role of socio-structural factors and psychosocial stress, including ongoing life-stressors related to socio-structural vulnerability, in excessive immune activation (Slavich and Sacher, 2019). These experiences have significant health consequences over the life-course, particularly when occurring in early life, including the development of mental health disorders such as depression (Mac Giollabhui, 2021; Kautz, 2021). Gender and sex-based differences have also been reported in depression and chronic inflammation outcomes. Evidence related to the biological mechanisms that underlie potential sex differences includes the influence of sex-hormones on inflammatory processes (Klein and Flanagan, 2016). There are also reported differences in biomarkers and depression symptom severity by sex-based indicators, with a greater burden (higher inflammatory biomarkers and greater severity of symptoms) among females (Labaka et al., 2018). Gendered differences in depression and inflammation, with higher reported rates among women, are attributed to psychosocial factors such as coping styles and strategies (e.g. active coping, rumination, seeking support, acceptance, avoidance), socialization, and differential experiences of social and structural stressors (Boyce et al., 2021; Schuch et al., 2014; Piccinelli and Wilkinson, 2000; Kuehner, 2017; Gough and Godde, 2018; Ernst et al., 2021; Niles et al., 2018).

For young people, the transition to adulthood brings a number of unique biological, immunological, and psychosocial challenges and changes, which can impact their health over the course of their life-time (Patel et al., 2007; Elgar et al., 2015). Despite this, there is a paucity of data on depression and inflammation among young people living in low-and-middle-income-countries (LMICs), who face a disproportionately higher risk of early onset depression and depressive symptoms, compared to their higher income country (HIC) counterparts (Pedersen et al., 2023). This differential risk is attributed to factors such as higher rates of poverty and economic deprivation, exposure to violence, frequent natural disasters, and a scarcity of accessible mental health services, all of which contribute to chronic stress that, over time, activates inflammatory responses and immune dysregulation (Beurel et al., 2020; Pedersen et al., 2023). Moreover, gender differences in this relationship remain underexplored, despite evidence of differential exposure to socio-structural stressors (Boyce et al., 2021; Gough and Godde, 2018; Ernst et al., 2021; Niles et al., 2018).

In South Africa, where this study is set, young people under the age of 29 make up just over half the population (Statistics South Africa, 2025). Research on the relationship between biomarkers of chronic stress, inflammation and mental health in South Africa is limited to data on adults and in the context of HIV research (Mudra Rakshasa-Loots et al., 2024; Thela et al., 2025; Sewpaul et al., 2019). Socio-economic inequities in post-Apartheid South Africa remain high (Umuhoza and Ataguba, 2018), which are likely contributors to the higher rates of lower mental health for youth, such as SES-related factors including income inequality, food insecurity, and experiences of violence (Link and Phelan, 1995; World Health Organization Commission on Social Determinants of Health, 2008; Buckley et al., 2020).

The primary objective of the study was to estimate associations between inflammation (measured via IL-6 and CRP) and probable depression among adolescents and young adults living in Soweto and Durban, South Africa. The secondary aim of this study was to determine if previously described risk factors, including gender and age, modify the effect of inflammation on depression. We hypothesized that individuals with elevated inflammation would have higher odds of depression.

## 2 Methods

### 2.1 Overview

This analysis used cross-sectional data, inclusive of survey (socio-demographic, mental health), clinical, and biological data from AYAZAZI (Kaida et al., 2018), a longitudinal cohort study (2014-2019) assessing intersecting socio-behavioural, structural, and biomedical HIV risk factors among South African adolescents and young adults. Data for the study was collected between 2014 and 2019, with participants enrolled across two study sites: the Perinatal HIV Research Unit (PHRU) located at Chris Hani Baragwanath Hospital in Soweto, Johannesburg, and the Wits Maternal, Adolescent and Child Health (MatCH) Research Unit (MRU), located at Commercial City centre in Durban.

To reflect gendered HIV prevalence in South Africa (Kaida et al., 2018), a recruitment target of 60% was set for young women to be enrolled in the study. Of the 425 study participants enrolled in the study, 220 were from Soweto and 205 from Durban. Participants were followed for 12 months in Durban and 18 months in Soweto, with regular follow-up visits every 6-months at both sites (Closson et al., 2019). Guided by a youth-engagement framework, the AYAZAZI study supported the meaningful inclusion of youth as valued partners in the research process (Dietrich et al., 2014).

#### 2.1.1 Study cohort and procedures

Participants were recruited through community outreach using posters, pamphlets, and word of mouth, as well as through the PHRU HIV Counselling and Testing clinic and a public sector reproductive health clinic located near the MRU site in Durban. Study inclusion criteria included residing in Soweto or Durban, being between 16 and 24 years of age, and self-reporting an HIV-negative or unknown HIV status. Participants were excluded if they were current participants in other clinical or observational HIV prevention studies.

Participants completed detailed socio-behavioural survey questionnaires, which were administered by trained young multilingual peer-interviewers in the preferred language of the participant (English, isiZulu, Sesotho) and completed online using DataFAX™ software. Survey questionnaires included questions assessing socio-demographics, sexual and reproductive health, experiences of violence, and mental health, and were reviewed by the adolescent community advisory board (CAB) at the PHRU. Biological and clinical data collection, which included pperipheral blood specimens, was performed at baseline and follow-up by study nurses or doctors present at each cohort site. Participants received a reimbursement of ZAR150 (approximately $12 USD) to compensate for travel costs and time spent at the study site, for each completed study visit. A detailed description of the survey and clinical data collection have been described elsewhere (Kaida et al., 2018; Closson et al., 2019).

### 2.2 Analytic sample

**Fig. 1:**
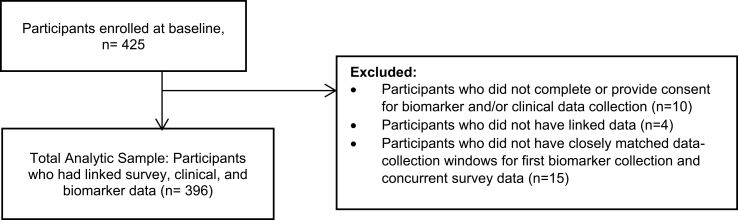
Analytic Sample flow-chart.

Our analytic sample consisted of all AYAZAZI study participants who provided survey, clinical, and immune marker data. We restricted the analytic sample to participants who provided survey, clinical, and immune data concurrently during the same data collection period (i.e. all data types were collected within the same 31-day time period) (n = 396) (Fig. 1). Given that not all participants had peripheral blood specimen collection at their baseline study visit, we used data from the first time point at which a participant provided concurrent, matched data. Among those who had complete, time-matched survey, clinical, and immune datasets, 332 (83.8%) participants provided this data at baseline data collection, while an additional 64 (16.2%) participants provided complete, time-matched data during the study's first follow-up visit (6-month follow-up) and were thus added to the analytic sample. Individuals were excluded from the analytic sample if their biomarker, clinical, or survey data were not collected within a 31-day timeframe.

### 2.3 Data

#### 2.3.1 Primary outcome

The 10-item Centre for Epidemiological Studies Depression (CES-D-10) Scale was used to assess depressive symptom frequency (past 7 days) with a CES-D-10 score of ≥10 indicating probable depression (Kilburn et al., 2018; Zhang et al., 2012). The scale uses a 4-point response format (“less than a day” = 0, “1-2 days” = 1, “3-4 days” = 2, “5-7 days” = 3), with possible scores ranging from 0 to 30. The scale has previously been validated in South Africa, indicating evidence for adequate construct validity and internal reliability (Baron et al., 2017), and has been used with youth living in Sub-Saharan African countries (Kilburn et al., 2018). Validation studies in South Africa indicate satisfactory reliability across diverse populations. A 2017 study by Baron et al., which included individuals aged 15 and over (median age 33), reported that the CES-D-10 had acceptable internal consistency across samples (α = 0.69–0.89), and adequate concurrent validity when compared with other depression scales. The authors did however reported that optimal cut-off scores for detecting significant depressive symptoms varied by ethnic group, indicating that cultural contexts may influence performance of the scale (Baron et al., 2017). Another validation study with 322 university students in the Western Cape province, South Africa, reported acceptable reliability indices (Padmanabhanunni and Pretorius, 2023). While the CES-D-10 has not been validated in an adolescent and young adult population comparable to AYAZAZI participants sociodemographically, South African (Baron et al., 2017; Padmanabhanunni and Pretorius, 2023) and regional data (Kilburn et al., 2018) suggest the scale can be an appropriate tool for use across age ranges in the South African context.

A cut off of ≥10 was used to indicate the presence of probable depression (significant depressive symptoms); this cut-off has previously been used and validated to differentiate between those who have significant depressive symptoms, and those who have no depressive symptoms or who have less significant depressive symptoms (Zhang et al., 2012; Andresen et al., 1994). Additional support, in the form of additional referrals and access to the study social worker, was provided for those who scored above the CES-D-10 threshold (≥10) for probable depression. Cronbach's alpha scores for the CES-D-10 were within the ‘acceptable’ range (0.72) (Tavakol and Dennick, 2011).

#### 2.3.2 Exposure: markers of inflammation

Inflammation was measured using the plasma biomarkers CRP, measured in mg/L, and IL-6, measured in pg/ml. Our choice of biomarkers CRP and IL-6 was driven by both biological hypothesis and strength of evidence, as they are both commonly used as well as biologically identified indicators of low-grade inflammation (Beurel et al., 2020; Osimo et al., 2019; Mac Giollabhui et al., 2021a) and immune dysregulation (Rodriquez et al., 2019), including in research with young people (D'Acunto et al., 2019; Colasanto et al., 2020). Peripheral blood specimens were collected from participants and processed on the same day at a nearby laboratory (Bio Analytical Research Corporation, in Soweto; HIV Pathogenesis Programme, University of KwaZulu-Natal, in Durban) to obtain plasma and stored frozen at −80 °C prior to analysis. Plasma specimens were analysed by Enzyme-Linked Immunosorbent Assay (ELISA) using the Meso Scale Diagnostics (MSD) human CRP V-PLEX and human IL-6 S-PLEX kits according to the manufacturer's recommendations. In our study, CRP concentrations were measured using the Meso Scale Discovery (MSD) Human CRP V-PLEX kit (K15198D), an electrochemiluminescence (ECL)-based immunoassay, while IL-6 was measured using the MSD Human IL-6 S-PLEX kit (K15396S), an ultrasensitive ECL assay platform (MESO SCALE DISCOVERY, 2022). The V-PLEX and S-PLEX kits are analytically validated by the manufacturer to provide accurate and reproducible results (MESO SCALE DISCOVERY, 2022). The lower limits of detection (LLOD) and quantification (LLOQ) were determined based on standard curves. For CRP and IL-6, all values were within quantifiable ranges. Intra-assay and inter-assay precision were assessed by calculating coefficients of variation (CV); mean CVs for both analytes were less than 10%. For CRP and IL-6, all values were within quantifiable ranges, and as such did not require imputation (Enzo Life Sciences, 2023). Analyte concentrations quantified on an MSD Quickplex 120 instrument using MSD Discovery Workbench software. Given the skewed nature of the data with the presence of significant outliers (Appendix), CRP and IL-6 values were natural log transformed to fit a normal distribution for regression analyses, and subsequently standardized to a mean of zero and standard deviation of one, to further reduce the potential influence of extreme values on regression estimates, and improve model interpretability. Biomarkers were treated as continuous measures in regression models, assuming a dose-response in the inflammation-depression relationship.

#### 2.3.3 Covariates

Covariate selection was informed by empirical evidence, and *a priori* hypothesized factors, based on a review of the literature (Arenas et al., 2019; Myers, 2020; Stubbs and Szoeke, 2022; Heath et al., 2013; Finegood et al., 2020; Yim and Kofman, 2019; O'Neil and Scovelle, 2018; Hamilton and Steptoe, 2023; Ribeiro et al., 2019; Blair et al., 2014; Schubert et al., 2017; Cotton and Shim, 2022; Rao et al., 2020), with causal diagrams (DAGs) used to inform model building (Appendix). Covariates were included as confounders in our regression models if they were a cause of the exposure, outcome, or both, and we considered proxies for unmeasured confounders of the outcome and exposure variables (VanderWeele, 2019).

Demographic covariates included age in years (16-24 years) as a continuous variable and dichotomized to convey the proportion of adolescents (16-18 years) and young adults (19-24 years) in the study. All participants identified as cisgender, meaning that their assigned sex at birth matched their reported gender identity, as such we chose to use the variable gender (men [reference]; women] in regression analyses. We included measures for monthly income ( ≤ ZAR 400 [reference]; ≥ ZAR 401), housing type (formal [reference]; informal [RDP (Reconstruction and Development Programme housing), shack, other]) and having financial dependents (no[reference]; yes). Food insecurity was assessed using the Household Hunger Scale (HHS), which measures house-hold food insecurity over the past 30 days; scores range from 0 to 6 (Deitchler et al., 2010). The HHS has previously been validated in various global settings, including Sub-Saharan Africa (Deitchler et al., 2010). For this analysis, the HHS was dichotomized, with ‘moderate or severe hunger’ (scores 2-6) indicating ‘yes’ and ‘little to no hunger’ (scores 0-1), indicating ‘no’. The study's use of the scale in the AYAZAZI study is described in greater detail in previous research (Jesson et al., 2021).

A substance use variable was created using combined data on alcohol and drug use (past 30 days), categorized as: no [reference]; yes. Alcohol use determined using three survey questions assessing use and frequency, measured binge drinking over the past month (no vs. yes), defined as 5 or more drinks in one day. To determine recent drug use in the past month [no; yes (to any)], participants who reported ever having used drugs on the survey were subsequently asked a series of separate questions (n = 16) about prescription (non-medical) and illicit drug use [i.e. heroin, cocaine, crack cocaine, lysergic acid diethylamide (LSD), ecstasy, methamphetamines (“tik”), Mandrax (methaqualone), nyaope (Mthembi et al., 2018), marijuana] in the past 30 days. We subsequently created a variable combining alcohol use and drug use over the past month (“substance use over the past 30 days”, no [reference]; yes). Smoking over the past 30 days was categorized as having smoked versus not over the past 30 days (no[reference]; yes).

Clinical data included lab-confirmed sexually transmitted infections (STIs); data collected was performed via nurse-collected vaginal swab specimens and physical examination. Nucleic acid amplification test (NAAT) methods were used to test for Chlamydia trachomatis (CT), Neisseria gonorrhoeae (NG), Mycoplasma genitalium (MG), and Trichomonas vaginalis (TV) (Kaida et al., 2018). Participants presenting with physical symptoms were further tested for Treponema pallidum, Haemophilus ducreyi and C. trachomatis Serovar L1-L3 via multiplex PCR using the Roche LightCycler. All participants underwent HIV testing using specific rapid test kits at scheduled study visits; in cases of positive rapid test results, further laboratory testing was conducted to confirm results. For the purpose of these analyses, clinically confirmed HIV status and seroconversion data were used.

### 2.4 Statistical analyses

Descriptive statistics, stratified by gender, summarized sociodemographic, health and behavioural characteristics of participants in the analytic sample, with bivariate analysis used to characterize mental health profiles according to established thresholds for the CES-D 10 scale. Logistic regression, crude and adjusted, was used to model associations between markers of chronic inflammation (serum CRP, IL-6 levels) and the outcome (probable depression) for the primary analysis. Confounders (n = 10) consisted of age, gender, income, housing category, financial dependents, food insecurity, substance use, smoking, HIV status, and presence of sexually transmitted infections (STIs).

For the secondary aim, interaction terms were included in logistic regression models to examine potential effect modification by gender and age on probable depression, guided by evidence supporting gendered and age-based variations in the inflammation and depression relationship (Mac Giollabhui et al., 2021b; Toenders et al., 2022). Complete case analysis for covariates of interest was used. All statistical analyses were carried out in R (version 4.1.3).

Missing Data: In total, 12 participants were missing data on either the outcome or covariates of interest. For those with missing data for either CES-D-10 (1-2 items missing data for 10-item scale; n = 8), or covariates of interest (food insecurity, n = 2; smoking, n = 2), we utilized multiple imputation to impute missing data. Little's MCAR Test (Little, 1988) was used determine if missing data in a dataset was missing completely at random (MCAR) or if there was a pattern to the missingness; operating under the missing at random (MAR) assumption, missing data in the analytic sample was imputed using the Multiple Imputation by Chained Equations (MICE) approach, using the [*mice*] package in R (Van Buuren and Groothuis-Oudshoorn, 2011; Rubin, 1987). Imputation models were constructed, creating 20 imputed datasets with 5 iterations. Regression analyses were performed on stacked imputed models; Rubin's rule was used to pool the imputed estimates (Rubin, 1987). A sensitivity analysis was performed with complete case data to compare outcomes with imputed data outcomes for the primary analysis.

### 2.5 Ethical considerations and consent procedures

The AYAZAZI study was granted ethical approval by the Research Ethics Boards in both Canada and South Africa from participating institutions, inclusive of Simon Fraser University, Canada (2014s0413), the University of the Witwatersrand, Johannesburg, South Africa ([HREC]–140707), and the University of KwaZulu-Natal (South Africa). At enrolment, study participants, aged 18–24 years, provided voluntary written informed consent. For younger participants aged 16–17 years, parents/legal guardians provided voluntary written informed consent, with voluntary written assent provided by the study participants; younger participants were re-consented if they reached 18 years of age during the study period. Bio-specimen collection required a separate consent process.

## 3 Results

### 3.1 Participant descriptives

Of the 396 participants included in the final analytic sample (Table 1), 49.7% (n = 197) were between the ages of 16-18 and approximately 59.3% (n = 235) were women. Overall, 42.7% (n = 169) had probable depression (49.8% women, 32.3% men; p < 0.001) at the start of follow-up; the median CES-D-10 score in the sample was 8 (5, 12). Food insecurity was experienced by 21.2% (n = 84) of study participants. In comparison to young men, young women were more likely to report living in informal housing (35.7% vs. 18.6%; p < 0.001), and have a lab diagnosed STI (24.7% vs. 8.1%; p < 0.001); young men were more likely to report drug or alcohol use in the past 30 days (73.9% vs. 39.1%; p < 0.001) and smoking cigarettes (43.5% vs. 15.3%; p < 0.001).

**Table 1:** AYAZAZI participant characteristics at start of follow-up, by gender (n = 396).

	Level	Overall, n = 396 n (%)	Men, n = 161 n (%)	Women, n = 235 n (%)	p-value
**Age category**	16 to 18	197 (49.7)	80 (49.7)	117 (49.8)	1.0
	19 to 24	199 (50.3)	81 (50.3)	118 (50.2)	
**Age (continuous)**	median (Q1, Q3)	19 (17, 20.25)	19 (18, 20)	19 (17, 21)	1.0
**Housing Type**	formal housing	282 (71.2)	131 (81.4)	151 (64.3)	<0.001
	informal housing	114 (28.8)	30 (18.6)	84 (35.7)	
**Monthly Income**	≤400 ZAR	91 (23.0)	43 (26.7)	48 (20.4)	0.118
	>400 ZAR	305 (77.0)	118 (73.3)	187 (79.6)	
**Financial Dependents**	none	285 (72.0)	122 (75.8)	163 (69.4)	0.200
	one or more	111 (28.0)	39 (24.2)	72 (30.6)	
**Food Insecurity**	not present	312 (78.8)	126 (78.3)	186 (79.1)	0.931
	present	84 (21.2)	35 (21.7)	49 (20.9)	
**Substance Use (past 30 days)**	no	185 (46.7)	42 (26.1)	143 (60.9)	<0.001
	yes	211 (53.3)	119 (73.9)	92 (39.1)	
**Smoking (past 30 days)**	did not smoke	290 (73.2)	91 (56.5)	199 (84.7)	<0.001
	smoke	106 (26.8)	70 (43.5)	36 (15.3)	
**STI lab diagnosis**	no	325 (82.1)	148 (91.9)	177 (75.3)	<0.001
	yes	71 (17.9)	13 (8.1)	58 (24.7)	
**HIV Status**	negative	384 (97.0)	158 (98.1)	226 (96.2)	0.411
	positive	12 (3.0)	3 (1.9)	9 (3.8)	
**Mental Health**					
**Probable Depression (CESD-10 ≥ 10)**	no	227 (57.3)	109 (67.7)	118 (50.2)	0.001
	yes	169 (42.7)	52 (32.3)	117 (49.8)	
**CES-D-10**	mean (SD)	9.17 (5.4)	8.01 (4.77)	9.96 (5.66)	0.005
	median (Q1, Q3)	8 (5, 12)	7 (4, 11)	9 (6, 14)	<0.001
**Inflammatory markers**					
**CRP, mg/L**	mean (SD)	4.13 (8)	2.60 (4.57)	5.18 (9.61)	0.002
	median (Q1, Q3)	1.70 (0.8, 4.2)	1.1 (0.6, 2.5)	2.2 (1.1, 5.1)	<0.001
**Elevated CRP (≥3.0 mg/L)**	no	263 (66.4)	126 (78.3)	137 (58.3)	<0.001
	yes	133 (33.6)	35 (21.7)	98 (41.7)	
**IL-6, pg/ml**	mean (SD)	1.74 (2.9)	1.32 (1.27)	2.03 (3.56)	0.015
	median (Q1, Q3)	1.11 (0.8, 1.8)	0.94 (0.7, 1.4)	1.31 (0.9, 2.1)	<0.001
**Elevated IL-6 (≥ 1.4 pg/ml)**	no	250 (63.1)	121 (75.2)	129 (54.9)	<0.001
	yes	146 (36.9)	40 (24.8)	106 (45.1)	

SD: standard deviation; Q1, Q3: 25th^,^ 75th percentile.

ZAR: South African Ran; Food Insecurity: measured using the Household Hunger Scale (HHS); Informal Housing: RDP (Reconstruction and Development Programme housing), shack, other; STI: Sexually transmitted Infection [inclusive of Chlamydia trachomatis (CT), Neisseria gonorrhoeae (NG), Mycoplasma genitalium (MG), Trichomonas vaginalis (TV), Treponema pallidum, Haemophilus ducreyi, C. trachomatis].

CES-D-10: 10-item Centre for Epidemiological Studies Depression Scale; Probable Depression: CES-D-10 Score ≥10; p-values were calculated using chi-square tests, with pooled values using Fisher's method for categorical variables, and Wilcox rank-sum tests with pooled values using Fischer's method for continuous.

Among young women, the median (Q1, Q3) plasma concentrations for CRP and IL-6 were 2.2 (1.1, 5.1) mg/L and 1.3 (0.9, 2.1) pg/mL, respectively. Among young men, median (Q1, Q3) serum concentrations for CRP and IL-6 were 1.1 (0.6, 2.5) mg/L and 0.94 (0.7, 1.4) pg/mL, respectively. A third of study participants (33.6%, n = 133) met the threshold for elevated CRP (≥3.0 mg/L), with a higher proportion among women compared with men (41.7% vs. 21.7%; p < 0.001); elevated IL-6 levels (≥1.4 pg/ml) were seen in 36.9% (n = 146) participants, with higher levels reported among young women (45.1% vs. 24.8%; p < 0.001).

### 3.2 Primary analysis: association between CRP, IL-6 and probable depression

In models using imputed data and controlling for covariates of interest (Table 2), CRP was associated with higher odds of probable depression in the overall sample in crude (OR 1.45; 95% CI 1.18-1.79), and adjusted models (aOR 1.32; 95% CI 1.06-1.66), where for each one standard deviation increase in log-transformed CRP, the odds of probable depression increased by 32%. While we found that per one-unit (standard deviation) increases in log-transformed IL-6 increased the odds of probable depression by 18% in unadjusted models, the results were not statistically significant at an alpha level of p < 0.05 (OR 1.18; 95% CI 0.96-1.44); in adjusted models the effects were further attenuated and not significant (aOR 1.06; 95% CI 0.85-1.32).

**Table 2:** Crude and adjusted odds ratios for the association between CRP and IL-6 and probable depression among AYAZAZI Study participants (n = 396).

Variable	Unadjusted		Adjusted	
	OR	95% CI	aORˆ	95% CI
**CRP**	1.45	1.18 – 1.79∗	1.32	1.06 – 1.66∗
**IL-6**	1.18	0.96 – 1.44	1.06	0.85– 1.32

aOR: adjusted odds ratio, 95% CI: 95% confidence interval; ∗p < 0.05.

**Adjusted for:** age, gender, income, housing category, financial dependents, food insecurity, substance use, smoking, sexually transmitted infections (STIs), HIV.

### 3.3 Secondary analysis: effect modification by gender & age

We conducted secondary analyses (Table 3) to examine possible effect modification of CRP and IL-6 on probable depression by gender and age. To do this, we included interaction terms between 1) CRP and gender, 2) CRP and age, 3) IL-6 and gender, and 4) IL-6 and age, in the adjusted logistic regression models. None of the interaction terms were statistically significant (all p > 0.05), which suggests that the associations between probable depression and CRP, and probable depression and IL-6, are independent of gender or age; however for CRP, the direction of effect on probable depression differed by gender, although this was not statistically significant.

**Table 3:** Adjusted odds ratios for the effect modification of gender, age, and markers of inflammation on depression among AYAZAZI study participants (n = 396).

CRP	aOR (95% CI)
*Gender∗CRP*	
Young men	REF
Young women	1.34 (0.84- 2.13)
*Age∗CRP*	
16-18 years old (adolescents)	REF
19-24 years old (young adults)	1.10 (0.72- 1.70)

IL-6	aOR (95% CI)
*Gender∗IL-6*	
Young men	REF
Young women	1.08 (0.67- 1.73)
*Age∗IL-6*	
16-18 years old (adolescents)	REF
19-24 years old (young adults)	0.69 (0.44- 1.07)

aOR: adjusted odds ratio, 95% CI: 95% confidence interval; ∗p < 0.05.

**Adjusted for:** age, gender, income, housing category, financial dependents, food insecurity, substance use, smoking, STI, HIV.

### 3.4 Sensitivity analyses: complete case regression for primary analysis

In a sensitivity analysis with complete case data (n = 384), CRP was significantly associated with probable depression, while the association with IL-6 remained non-significant. In the unadjusted model, the complete case data showed a significantly increased odds of depression for CRP (OR 1.36; 95% CI 1.10-1.67), as well as for IL-6 (OR 1.24; 95% CI 1.00, 1.52) (Table 4). Controlling for covariates of interest, CRP was associated with higher odds of probable depression in the overall sample in crude adjusted models (aOR 1.25; 95% CI 1.00-1.57). There was a positive, though not significant (p > 0.05) association between IL-6 and probable depression in adjusted models with complete case data (aOR 1.16; 95% CI 0.92 – 1.46).

**Table 4:** Crude and adjusted odds ratios for the association between CRP and IL-6 and depression among AYAZAZI Study participants, using complete case data (n = 384).

Variable	Unadjusted		Adjusted	
	OR	95% CI	aORˆ	95% CI
**CRP**	1.36	1.10 – 1.67∗	1.25	1.00 – 1.57∗
**IL-6**	1.24	1.00 – 1.52∗	1.16	0.92 – 1.46

aOR: adjusted odds ratio, 95% CI: 95% confidence interval; ∗p < 0.05.

**Adjusted for**: age, gender, income, housing category, financial dependents, food insecurity, substance use, smoking, STI, HIV.

## 4 Discussion

In this cross-sectional component of adolescents and young adults in South Africa, we found that higher serum concentrations of CRP were significantly associated with the presence of significant depressive symptoms or probable depression, with a 32% increased odds of probable depression per each standard deviation increase in CRP (mg/L). While we saw a positive relationship for IL-6 and probable depression (aOR 1.07), the association was not statistically significant. Our findings align with a growing body of evidence linking higher levels of inflammatory biomarkers with probable depression among youth (Mac Giollabhui et al., 2021a), and provide important, supporting evidence to the investigation of the inflammation-depression relationship among adolescents and young adults living in LMICs.

To contextualize our findings and their clinical significance, a 32% higher odds of probable depression in our sample associated with each standard deviation increase in log-transformed CRP, represents a significant change from approximately 1.93 mg/L to 6.35 mg/L in the original raw data. We interpret this to indicate a significant shift in the inflammatory burden, given that the low-moderate inflammatory risk threshold of 1-3 mg/L as reported in the literature on chronic illnesses (Calder et al., 2013; Nehring et al., 2017). The association of this increase with a 32% higher odds of depression is consistent with neuroinflammatory pathways discussed in the literature on depression and inflammation (Beurel et al., 2020; Mac Giollabhui, 2021), and is consistent with the direction and magnitude of associations that have been reported by other studies with youth and adults globally (Haapakoski et al., 2015; Mac Giollabhui et al., 2021a; Colasanto et al., 2020).

Previous studies have consistently found elevated levels of inflammatory biomarkers in individuals with major depressive disorder (Beurel et al., 2020; Haapakoski et al., 2015; Osimo et al., 2019), as well as among youth reporting significant depressive symptoms, suggesting that inflammatory proteins and cytokines may contribute to depression sub-types and symptom profiles (Beurel et al., 2020). Depression subtypes are characterized by variations in duration, trajectories, and symptom features, and as well as differences in biological system dysregulation, which all contribute to depression heterogeneity including treatment response (Beijers et al., 2019). Research examining mechanistic pathways between inflammation and mental health outcomes indicates that higher levels of biomarkers such as CRP may reflect a chronic inflammatory state. CRP is a down-stream inflammatory marker primarily induced by IL-6-dependent hepatic biosynthesis, which mediates acute-phase responses following an inflammatory event or stimulus. It is involved in both pro and anti-inflammatory processes of both chronic and acute immune system response (Calder et al., 2013; Nehring et al., 2017). CRP activity may influence mental health through multiple neurobiological mechanisms, including altered neurotransmitter activity, metabolism and availability as well as immune and stress-response dysregulation (Beurel et al., 2020). Furthermore, other studies have also shown that induction of inflammatory responses, which correspond to measurable elevations in inflammatory biomarkers, can bring on depression-related symptoms and behaviours including fatigue, low mood, and anhedonia (Liu et al., 2017; Lasselin et al., 2021; Cho et al., 2019). Such findings support hypotheses that inflammatory factors may not only correlate with depression but contribute to its onset (Dantzer et al., 2007; Slavich and Irwin, 2014a; Slavich et al., 2010).

Our null findings for IL-6 and probable depression may point to both biological and methodological reasons. While IL-6 is a widely studied and consistently associated biomarker for depression (Mac Giollabhui et al., 2021a; D'Acunto et al., 2019; Köhler et al., 2017), its biological function as both an anti-inflammatory and pro-inflammatory cytokine, as well as the proposed pathways through which it impacts depression (Tanaka and Kishimoto, 2014; Ferrucci et al., 2005; Köhler et al., 2017), likely influence on the results. Research indicates elevated levels of IL-6 are most commonly associated with depression, preferencing the pro-inflammatory, trans-signaling pathway through which IL-6 has an impact on a wide range of cells promoting immune responses and stimulating the production of other inflammatory biomarkers, as well as disrupting the HPA axis (Köhler et al., 2017). However, other biological mechanisms may be at work, and could point to differences in depression subtypes (Köhler et al., 2017). Furthermore, not all individuals with depression exhibit increased inflammation, and varied types of inflammatory markers are associated with different types of inflammatory markers (Beurel et al., 2020; Dunjic-Kostic et al., 2013). The consideration of timing and temporal fluctuations may play a role in IL-6 and depression associations, as would confounding factors including comorbidities, assay methodology (Beurel et al., 2020), and statistical power to detect an effect.

In South Africa, health inequities remain prevalent in the post-apartheid years, indicating enduring effects of colonial and segregationist policies (Scott et al., 2017; Omotoso and Koch, 2018; Mayosi et al., 2012; Statistics South Africa, 2020). Evidence shows that societal inequities shape health through persistent and racialized socioeconomic inequalities, barriers to care access and other recourses, and high rates of violence and lower community safety, which have shown persisting inequities, including HIV risk, high rates of mental health concerns as well as differences in growth and development, particularly among young women (Omotoso and Koch, 2018; Richter et al., 2007, 2018). South African studies with both adult and youth populations have consistently found strong associations with social determinants such as exposure to violence, low socio economic status, food insecurity, indicating a profound and interconnected burden of common mental health issues and SDOH inequities (Jesson et al., 2021; Mngoma et al., 2021; Mungai and Bayat, 2019). Associations with social determinants of health are present across studies for higher-income countries, indicating that socio-economic and other structural inequities play a consistent, important role in impacting the inflammation and depression (Lund et al., 2018; Slavich and Irwin, 2014b; Muscatell et al., 2020).

Despite evidence of an inflammation-depression relationship (Valkanova et al., 2013; Mac Giollabhui et al., 2021a), the directionality of the inflammation-depression relationship remains unclear. Some studies suggest that inflammation precedes and predicts depressive symptoms, while others indicate that depression itself may lead to inflammatory responses (Beurel et al., 2020; Valkanova et al., 2013; Mac Giollabhui et al., 2021a; Berk et al., 2013), where depression-related behaviours, such as social withdrawal, poor sleep, and altered health behaviours, may exacerbate immune activation, creating a feedback loop contributing to excessive immune activation and dysregulation (Liu and Alloy, 2010; Hammen, 2005). Given these complexities, additional longitudinal studies are needed to disentangle causal pathways, particularly studies that account for sex/gender disaggregated analysis and control for confounding factors. Additionally, expanding measures of chronic inflammation (beyond CRP and IL-6 alone) (Valkanova et al., 2013; Mac Giollabhui et al., 2021a) could help better elucidate processes around the role of chronic inflammation in depression and biological consequences of social stress (Logie et al., 2022).

Although we did not find a significant impact of sex/gender on CRP's relationship with probable depression, we observed higher, non-significant, odds of depression for higher CRP among female participants. Previous research suggests that there may be gendered differences in associations for CRP and depression (Mac Giollabhui et al., 2021b). Gender and sex influences are complex, multifactorial, and include biological and social factors, including differential experiences of psychosocial stress (Boyce et al., 2021; Gough and Godde, 2018; Ernst et al., 2021; Niles et al., 2018). Studies examining inflammation and depression have produced mixed results, with some finding stronger associations between inflammation and depression for women (Zainal and Newman, 2021; Kohler-Forsberg et al., 2017), while other studies found stronger associations among men (Liukkonen et al., 2006; Lee and Min, 2023). These mixed results may indicate life-course fluctuations and changes in both immune function (Klein and Flanagan, 2016) and depression rates (Villarroel and Terlizzi, 2019), or may point to distinct pathways to depression, with variations resulting from social and/or biological mechanisms related to sex and gender differences (as well as their interactions). For example, research indicates that hormones (and hormonal fluctuations at different life-periods including during puberty), as well as socio-behavioural and biological factors may play a larger role in predicting depression among women (Ernst et al., 2021; Mac Giollabhui et al., 2021b; Liukkonen et al., 2006; Liu et al., 2014; Jha et al., 2018), and thus may require further exploration and disentanglement, with specific exploration of sex and gender pathways and variable consideration. Nevertheless, it can be particularly difficult to distinguish or clearly disentangle sex and gender, as they are neither binary nor mutually exclusive, with evidence that biological and environmental/social factors can and do interact to affect risk (Kuehner, 2017; Springer et al., 2012). While our results do not align with other research on sex and gender differences, our findings may be reflective of other factors which may play a stronger role in impacting the relationship between chronic inflammation and lowered depression. Furthermore, given our small sample size, our models may not have been sufficiently powered to detect significant associations in the variability of the inflammation-depression relationship by gender or age, and as such better-powered studies may be useful to detecting sub-group differences in these associations.

Though not statistically significant in our study, the relationship between depression and inflammation in youth have been reported in other studies. A meta-analysis focused on youth reported significant associations between depression and CRP, though trends did not appear to differ significantly between the ages studied (Colasanto et al., 2020). Despite limitations in the data and mixed results, findings from several studies demonstrate that early life inflammation can be an important factor in predicting depressive symptoms in youth, over large follow-up periods (Walss-Bass et al., 2018; Chu et al., 2019; Khandaker et al., 2018), providing additional support to developmental paradigms related to mental health (Boyce et al., 2021). Thus, a greater and intersectional exploration of additional socio-structural factors may be helpful in parsing out whether differential pathways to depression exist for men and women, and how these factors may further mediate pathways among youth and young adults.

### 4.1 Strengths and limitations

Our study has several strengths and limitations. The cross-sectional nature of the data included in this analysis precludes the opportunity to determine temporality, limiting causal inference. However, determining cause and effect is supported by research indicating that inflammatory markers represent cumulative exposures (Valkanova et al., 2013), whereas depressive symptoms, being commonly episodic in nature (Malhi and Mann, 2018) were assumed as current to the time of study enrolment. Probable depression in our study captured recent depressive symptoms (experienced over the past seven days), which may not represent a true relationship with more chronic inflammatory activity overtime. Thus, given that depression is an episodic illness, longitudinal measures of inflammatory biomarkers (e.g. CRP and IL-6) and depression outcomes would be useful to disentangling directionality and causal associations (van den Biggelaar et al., 2007; Stewart et al., 2009).

While the CES-D-10 is a previously validated screening tool, including in South Africa with similar populations, it is a screening tool measuring depressive symptoms over the past two weeks, and does not provide a clinical diagnosis. A clinical diagnoses of major depressive disorder would provide more detailed information on depression type, which may be useful for untangling the complex relationship between inflammation and depression subtypes (Beurel et al., 2020). Nevertheless, while a score of ≥10 on the CES-D 10 doesn't equate to a formal diagnosis of major depressive disorder as outlined in the Diagnostic and Statistical Manual of Mental Disorders, fifth edition (DSM-V) (American Psychiatric Association, 2013), studies examining its diagnostic precision and criterion validity have confirmed that the CES-D-10 is reliable tool for identifying clinically significant depressive symptoms in youth (James et al., 2020). Given its self-reported nature, though more susceptible to bias, its use does not require clinician administration, making it an appropriate tool for epidemiologic research, including in lower-resource settings, as an initial screening tool for the presence of significant depressive symptoms (Ramdhonee‐Dowlot et al., 2025).

Due to some participants not providing biological sample data at their first, baseline study visit, our cross-sectional analytic sample was created using each participants first biomarker visit data linked with concurrent survey and clinical data. The addition of 64 individuals represents a significant increase in sample size, providing a meaningful improvement in the analytic capacity of the study, given that statistical power is a function of sample size, effect size, and significance threshold (Cohen, 2016). Given potential for biological variability in biomarker levels (Haapakoski et al., 2015; Köhler et al., 2017), including within-person for some biomarkers, an increase in sample size reduces the risk of missing the presence of a significant association between IL-6 or CRP and depression, and strengthens our analysis. We also note that given that probable depression decreased over time in our study (Pakhomova et al., 2025), we acknowledge that the inclusion of individuals with survey data at their 6-month follow up visit (n = 64) may have biased the results, considering that study participation may have influenced longitudinal depressive symptoms trends, given our employment of a youth engagement framework in the design and implementation of the study which provided youth centered spaces and additional supports for those experiencing significant depressive symptoms. However, sensitivity analyses with true baseline participants (n = 332) data did not yield significant differences in effect estimates, or changes in the direction of effects.

Unmeasured confounders may have influenced our findings, including prior history of depression, early life adversity, or a robust measure of socioeconomic inequity, which could contribute to residual confounding potentially biasing the results. While BMI was initially considered as a possible covariate, we chose not to include it as it may not adequately capture the adiposity-related inflammatory burden in South African youth, given that cut-offs were established in Western Populations and thus may not appropriately measure adiposity associated risks in this population, potentially increasing the risk of residual confounding (Maydych, 2019).

Future longitudinal analyses of the AYAZAZI study data will be helpful in unravelling the pathways between inflammation and depression among this cohort. While CRP and IL-6 are well studied measures of chronic inflammation, future studies could benefit from incorporating composite scores consisting of other relevant biomarkers for chronic inflammation (Beurel et al., 2020) to better understand and parse out how inflammation affects mood and mental health. CRP is implicated in the etiology of depression, however its function as marker of acute infection can impact the interpretability of results. We do acknowledge this limitation to the scope of CRP or IL-6 as appropriate markers of chronic inflammation etiologically important to depression, and that inflammation may not be a causal factor in all cases of depression (Valkanova et al., 2013; Mac Giollabhui et al., 2021a). However, unresolved inflammation can lead to dysregulation of immune function, resulting in persistent low-grade stimulation of inflammatory responses such as the over-production and continuing presence of acute inflammatory proteins such as cytokines and acute phrase proteins like CRP (Furman et al., 2019; Maydych, 2019; Dowlati et al., 2010). Given the heterogeneity of depression/depression sub-groups (Beurel et al., 2020), other markers of inflammation may be relevant and should be explored in future studies. While other biomarkers appear in the literature as potential markers of chronic inflammation and are linked with depressive symptoms, the evidence is most consistent for IL-6 and CRP, which have the most robust and consistent associations with depression (Haapakoski et al., 2015; Osimo et al., 2019; Valkanova et al., 2013; Strawbridge et al., 2017). We do however acknowledge that for young people, evidence is less consistent for CRP and IL-6 (Toenders et al., 2022), and posit that future research could benefit from a more exploratory examination of a wider range of biomarkers. We also note that while there may be a potential for a Type 1 error in our results, each of our independent models were specified *a priori* and supported by a substantial evidence base on markers of inflammation and depression outcomes, rather than exploratory in nature. We also note that differing findings for CRP and IL-6 are biologically plausible (Tanaka and Kishimoto, 2014; Ferrucci et al., 2005; Köhler et al., 2017).

Finaly, while youth-engagement methods included peer-interview assistance with completion of the survey tools, allowing for participants and interviewers to build rapport, the reporting of sensitive data (e.g. sexual and reproductive health, experiences and perpetration of violence) in the presence of peers may have introduced social desirability and recall bias.

## 5 Conclusion

CRP was significantly associated with greater odds of probable depression in our cohort of young South Africans. No statistically significant associations were found for IL-6, or gender and age-modified analyses. The findings from our study align with a growing body of evidence linking higher levels of CRP with depression among youth, adding valuable insights into understanding the inflammation-depression relationship among youth in high-stress environments. These results have important implications both for future research as well as prevention strategies, including interventions which focus on upstream, socio-structurally focused strategies to address underlying conditions which may drive both inflammatory and mental health vulnerability in youth living in LMICs. Longitudinal studies can present an important avenue for examining causal associations and the impact of other social and structural factors, as relevant to determining temporal trends and mechanisms in disease etiology. This study will inform next steps in investigating the inflammation-depression longitudinal relationship among youth to help determine variability in pathways of inflammation-depression.

## Funding

The AYAZAZI Study was funded through the Canadian HIV Vaccine Initiative (CHVI) and the Canadian Institutes of Health Research (CIHR), with support from the South African Medical Research Council (SAMRC). JJ Dietrich was supported by the Early Career Investigators Programme through SAMRC's Research Capacity Development Initiative, with funding received from the South African National Treasury. TN was partially supported through the Sub-Saharan African Network for TB/HIV Research Excellence (SANTHE) which is funded by the Science for Africa Foundation [Del-22-007] with support from Wellcome Trust and the UK Foreign, Commonwealth & Development Office and is part of the EDCPT2 programme supported by the European Union; the Bill & Melinda Gates Foundation [INV-033558]; and Gilead Sciences Inc. [19275]. The content and findings reported and illustrated are the sole deduction, view and responsibility of the researchers and do not reflect the official position and sentiments of the SAMRC or any of the funders.

## CRediT authorship contribution statement

**Tatiana E. Pakhomova:** Conceptualization, Formal analysis, Investigation, Methodology, Software, Visualization, Writing – original draft, Writing – review & editing. **Thumbi Ndung'u:** Funding acquisition, Investigation, Resources, Writing – review & editing. **Mark Brockman:** Funding acquisition, Investigation, Resources, Writing – review & editing. **Anne Gadermann:** Supervision, Writing – review & editing. **T.J. Salway:** Supervision, Writing – review & editing. **Mags Beksinska:** Funding acquisition, Investigation, Resources, Writing – review & editing. **Amanda Rowlands:** Writing – review & editing. **Kalysha Closson:** Writing – review & editing. **Julie Jesson:** Writing – review & editing. **Stepfanie Vermaak:** Project administration, Writing – review & editing. **Smritee Dabee:** Writing – review & editing. **Janan J. Dietrich:** Funding acquisition, Investigation, Resources, Writing – review & editing. **Jenni Smit:** Funding acquisition, Investigation, Resources, Writing – review & editing. **Mzikazi Nduna:** Writing – review & editing. **Angela Kaida:** Funding acquisition, Investigation, Resources, Supervision, Writing – review & editing.

## Declaration of competing interest

The authors declare that they have no known competing financial interests or personal relationships that could have appeared to influence the work reported in this paper.

## Data Availability

Due to the sensitive nature of the questions asked in this study, data cannot be shared publicly. For researchers and trainees who meet criteria for accessing confidential study data, requests can be sent to the corresponding author, Dr. Angela Kaida, at kangela@sfu.ca. Criteria for accessing confidential data includes being added as an AYAZAZI researcher or trainee to the SFU research ethics board application and signing the AYAZAZI Data Sharing and Collaboration Agreement. Co-authorship is a requirement for data access. The de-identified dataset cannot be publicly shared as we do not have community or REB approval to do so. Similarly, please contact the corresponding author, Dr. Angela Kaida, to request access to the questionnaires used in the AYAZAZI study.
