# Supplementary material for: Associations between inflammation markers (CRP and IL-6) and depression among adolescents and young adults in Soweto and Durban, South Africa
Source: Brain Behav Immun Health. 2026 May 19;55:101265. doi: 10.1016/j.bbih.2026.101265 (PMC13312184; doi:10.1016/j.bbih.2026.101265)
Supplement: Multimedia component 1 [file mmc1.docx]

**APPENDIX**

**Figure A: Distribution of raw CRP values among AYAZAZI study participants (n=396)**


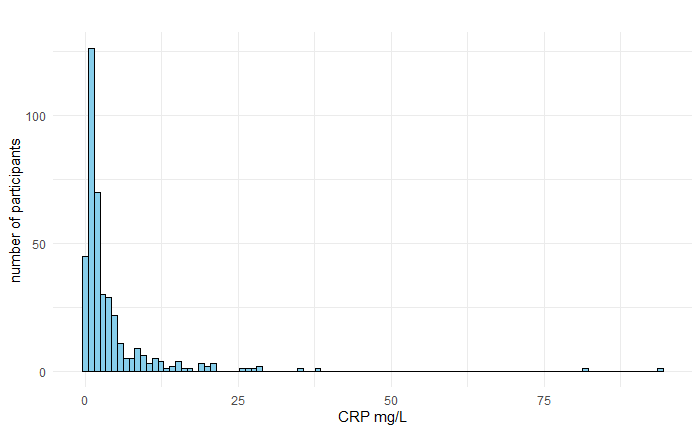


**Figure B: Distribution of raw IL-6 values among AYAZAZI study participants (n=396)**


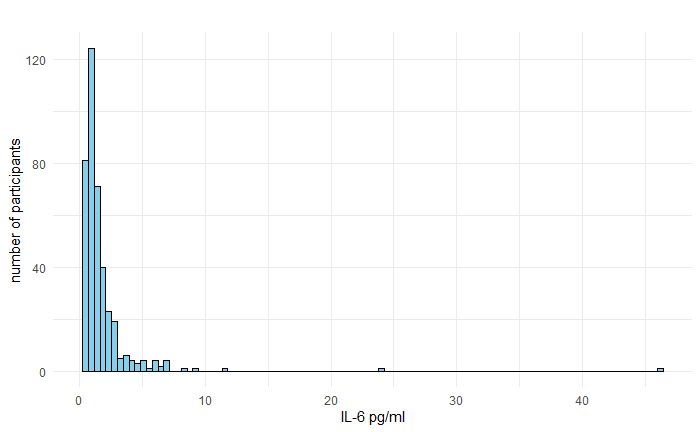


**
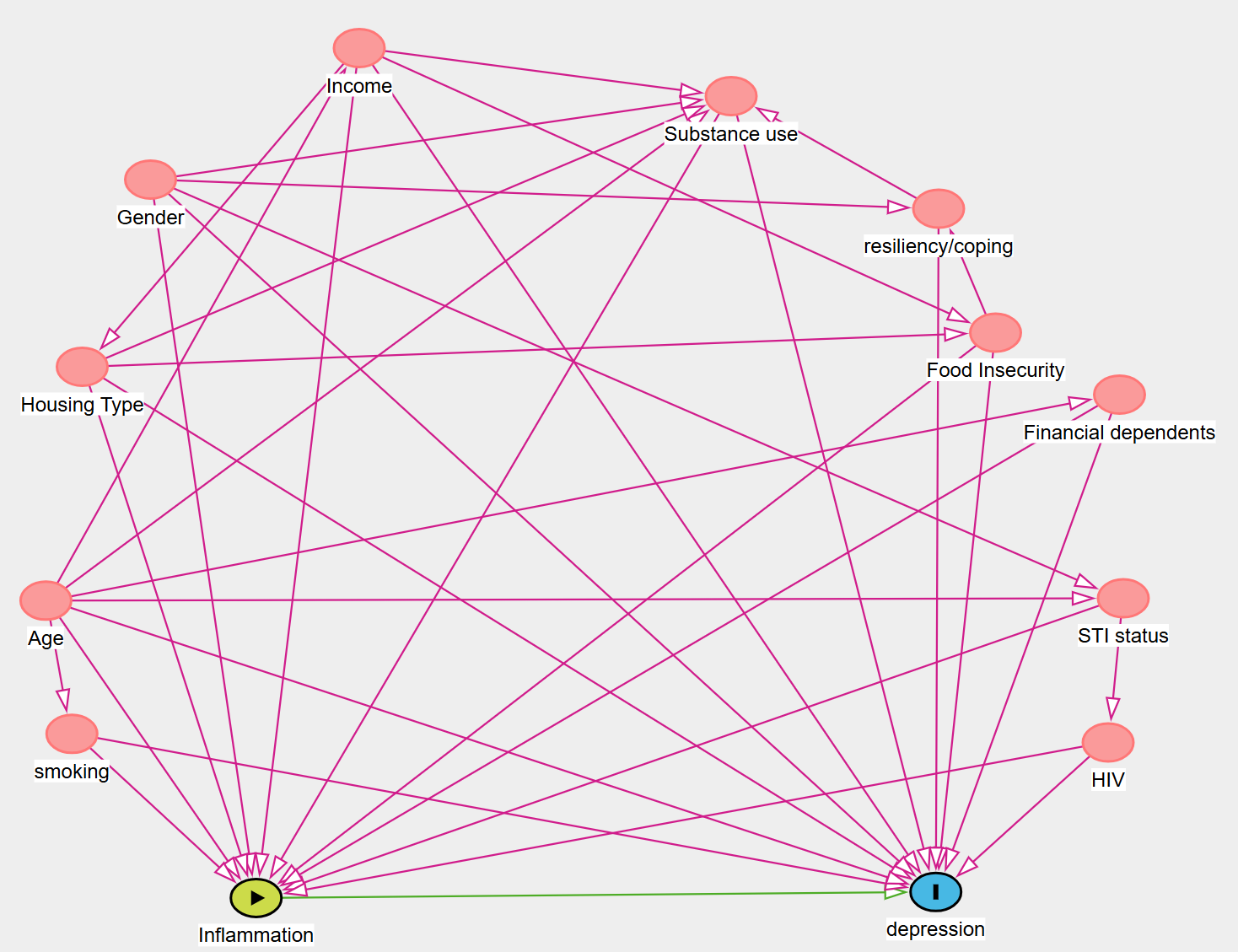
**

**Figure C:** Directed Acyclic Graph representing the relationship between inflammation and depression, as well as relevant confounders. Minimal Sufficient Adjustment set includes: Age, Financial Dependents, Food Insecurity, Gender, HIV, Housing Status, Income, STI status, Substance use, Smoking
